# Reimagining “racial” stratification and inequality: inter- and intraracial colorism in Trinidad and Tobago

**DOI:** 10.3389/fsoc.2025.1716832

**Published:** 2026-01-16

**Authors:** Monique D. A. Kelly

**Affiliations:** Department of Sociology, Michigan State University, East Lansing, MI, United States

**Keywords:** Anglophone Caribbean, colorism, educational attainment, household amenities, skin shade stratification, Trinidad and Tobago

## Abstract

**Introduction:**

Research on ethnoracial inequality in Trinidad and Tobago has principally focused on intergroup comparisons using broad census categories to examine differential access to key outcomes. Fewer studies, however, have examined how colorism—the systemic conferral of (dis)advantages based on one’s rank on a skin shade gradient—shapes life chances. Using skin shade, an embodied cue used in the ascription of race, may offer more nuanced and comprehensive understandings of inequality. Conceptualizing colorism as a continuum bounded both between and within racialized groups, this study offers a unique lens on how skin shade structures socioeconomic outcomes.

**Method:**

Using nationally representative data from the 2010–2023 AmericasBarometer surveys, I examine the impact of interviewer-rater skin color on two key indicators of socioeconomic wellbeing—educational attainment and relative wealth—both inter- and intraracially. I also assess the relative effects of intraracial colorism by ethnoracial group.

**Results:**

Findings show that darker skin is significantly associated with reduced odds of attaining higher levels of education and reduced access to household amenities. Intraracially, color-based disparities persist across all groups: East Indians are most affected in terms of educational access, while mixed-race individuals show the largest disparities in household amenities.

**Discussion:**

The study highlights the multidimensionality of colorism by integrating intraracial and interracial analyses. By centering the case of Trinidad and Tobago, the findings highlight the enduring power of embodied cues—i.e., skin shade—in structuring social outcomes. Moreover, this study emphasizes the need for increased recognition of colorism as an active and salient racialized stratifier that shapes life chances, apart from more commonly centered “racial” divisions.

## 1 Introduction

Research on racialized inequality has primarily focused on intergroup comparisons, often positioning individuals within constrained socially constructed “ethnoracial” categories (Monk, 2022; Strmic-Pawl et al., 2021). These categories often group individuals based on perceived commonalties of ancestry, language, religion, and/or geographical origin (Dixon and Telles, 2017). However, such nominal categories often obscure heterogeneity within groups, blur commonalities across them, and flatten gradational differences in racialized social experiences. This framework also cannot account for embodied traits, such as skin shade, that serve as visible cues used to assign ethnoracial typicality (Banton, 2012; Monk, 2022; Wade, 2012). Although ethnoracial determinations based on skin shade gradations are oftentimes coupled with other phenotypic characteristics, like facial features and hair type, skin shade remains a central cue in these appraisals (Feliciano, 2016; Flores and Telles, 2012) and drives cognitive judgments connected to group based stereotypes (Jablonski, 2021; Maddox, 2004; Maddox et al., 2022).

Previous research has consistently shown that skin shade operates as a distinct and salient stratifier, that functions as a form of embodied capital that corresponds to material benefits (Ellis and Destine, 2023; Hunter, 2007, 2021; Monk et al., 2021). The social and economic capital gained from the embodied trait of skin shade is referred to as colorism—the unequal distribution of material (dis)advantages along a color continuum (Hunter, 2007; Monk, 2021a; Tate, 2007). In fact, studies frequently find that lighter skin shade is often associated with systemic advantages across domains such as socioeconomic status, health, partner selection, and judicial treatment, though exceptions exist (Adames, 2023; Hall, 2022; Hall and Mishra, 2024; Monk, 2016, 2021b; Painter et al., 2019).

Centering phenotypic cues in the production of racialized stratification is therefore essential (Monk, 2022). By investigating how social perceptions and context shape the impact of colorism on racialized groups, scholars can more effectively identify the underlying dynamics through which skin shade stratification operates (Dias, 2020; Hordge-Freeman, 2015). The (dis)advantages associated with skin shade unfold within the symbolic boundaries of contextual ethnoracial commonsense. That is, based on a society’s specific history and dynamics of racial formation (Omi and Winant, 2018), the effects of colorism may vary based on how skin shade maps onto presumed prototypical features of ethnoracial categories in each society (Cartwright, 2022; Pedulla, 2018). Taken together, this body of work suggests that examining the impacts of skin shade on life outcomes not only reveal forms of inequality that might otherwise remain obscured but also provides a more precise, comprehensive, and theoretically robust lens for examining the reproduction of social inequality than census-based categories alone.

Although extensive, much of the literature on colorism has emphasized either interracial or intraracial dynamics in isolation, with few exceptions (e.g., Barlow et al., 2025; Branigan et al., 2017; Painter and Holmes, 2023). This focus leaves both an empirical and theoretical gap in our understanding of how perceived ethnoracial typicality and skin shade stratification vary among racialized groups across contexts. By comparing how colorism operates both between- and within-groups, this dual approach highlights its multidimensional nature (Dixon and Telles, 2017; Monk, 2022; Strmic-Pawl et al., 2021) and reveals variations in skin shade stratification across social outcomes. To address these gaps, I center the case of Trinidad and Tobago and draw on data from the AmericasBarometer to examine the association between interviewer-rated skin color and two key indicators of socioeconomic wellbeing, both interracially and intraracially. I then assess the relative effects of colorism within each ethnoracial group on these outcomes.

Trinidad and Tobago is both theoretically and empirically significant for several reasons. First, it fills a critical empirical and theoretical gap by providing quantitative analysis of colorism and its structural impacts in the Anglophone Caribbean—an underexplored region where such work remains scarce. Second, the country’s substantial ethnoracial diversity (Central International Agency, 2025) offers a unique opportunity to examine stratification outside of a U.S.-centric Black–White binary, particularly among intermediate ethnoracial groups. This illustrates how pigmentocratic hierarchies can persist in a majority non-White society, while also challenging assumptions rooted in the region’s tripolar racialized hierarchy of White, mixed-race, and Black.

Third, the study advances understanding of how skin shade structures inequality by situating colorism with Trinidad and Tobago’s racialized past. Colonial policy often pitted East Indian indentured servants against emancipated West Africans (Munasinghe, 2018; Reddy, 2011; Williams, 1962) and the consequences of such policies and subsequent ethnoracial tensions are well documented (Brereton, 2010; Gosine, 1987; Rakhal-Fraser, 2022a, 2022b; Rampersad, 2022; Ryan et al., 1979; Stone, 1972). Literature on racial inequality has thus focused on disparities between Black (Afro-) and East Indian (Indo-) Trinbagonians (Coppin and Olsen, 1998; Premdas and Bangura, 2006; Schimanski et al., 2018; Sriskandarajah, 2005). As a result, our understanding of how skin shade influences social outcomes remains limited (e.g., Kelly, 2022; Khadan and Ruprah, 2024; McCree, 2015; Painter et al., 2019), and within group distinctions remain relatively understudied, often focusing narrowly on Afro- and/or Indo-Trinbagonians (e.g., Doubeni, 2017; Elwin, 2022; Khan, 2009; Rampersad, 2012).

Additionally, the country has a large mixed-race population, whom historically have had greater access to wealth, education, and skill attainment, along with greater chances of being manumitted compared to many mono-racial and darker skinned Black people. As lighter skin shade was often used as a marker of racial admixture during enslavement (McCree, 2015; Stewart, 2004; Williams, 1962), color has long played a central role in racialized stratification. Fourth, by situating Trinidad and Tobago within broader sociological debates, the study enriches the field of social stratification by expanding the geographic and contextual scope of research, demonstrating how insights from Trinidad and Tobago can complicate and expand sociological theorization.

### 1.1 Theoretical framework

Monk (2022) presents an Infracategorical Model of Inequality (ICMI) as a means to move beyond reliance on state categories, which often limit analytic clarity and nuanced comprehension, to broaden our understanding of social inequality. The model therefore shifts attention from nominal group membership to the embodied cues of typicality that shape classification. Thus, emphasizes disaggregating differences by centering the body itself (i.e., bodily capital; see Ellis and Destine, 2023; Hordge-Freeman, 2015; Hunter, 2011; Monk et al., 2021), which signals social status and group belonging. To this end, Monk (2022) argues that examining colorism offers a valuable alternative lens with which to examine racialized inequality as differences in social outcomes observed between nominal ethnoracial groups often vary by skin shade. For example, research on colorism has shown that among African Americans, within-group disparities are sometimes greater than between-group disparities (Keith and Herring, 1991; Monk, 2015). Examining skin shade stratification thus offers critical insights into enduring questions of racial inequality. In doing so, the ICMI draws attention to the often-overlooked processes of social perception in structuring inequality yet carries little sociopolitical salience.

The extensive evidence provided by Monk (2022) affirms the existence of within-group inequality based on factors that shape ethnoracial prototypicality. This work highlights how skin shade, as a form of bodily capital, influences the racialization of individuals in specific contexts—such as the United States in Monk’s case—and how these processes are reinforced through institutions. In many postcolonial societies, lighter skin is especially advantageous because it signifies closeness to Whiteness (or White men) (Livesay, 2018; Sio, 1976). This proximity not only reflects colonial histories but also elevates individuals within racialized hierarchies based on their skin shade. Scholars have similarly conceptualized Whiteness itself as a form of bodily capital that includes lighter skin shade as well as other physical and linguistic features (Adames, 2023; Delgado, 2018). The degree of bodily capital ascribed to a person can then be converted into other forms of capital, such as social and economic resources (Hunter, 2002; Monk et al., 2021).

Due to ethnoracial classifications produced through colonialism, enslavement, indentured labor, and migration, lighter skin shades may signal intermediary status within the Americas’ hierarchal racialized field of White supremacy and anti-Blackness.^1^ Therefore, the value and conversion of lighter-skin bodily capital, as well as the severity of colorism, depend on the racialized group in question, the context of racialization, and the extent to which skin shade is perceived to affirm or violate local ethnoracial typicality.

In societies within Latin America and the Caribbean, race is not conceptualized as rigidly as in the U.S. (Davenport, 2020; Dixon and Telles, 2017), as such, there is often greater room for interracial differentiation. For example, research on racial fluidity—the socio-contextual malleability of race (Davenport, 2020; Telles and Paschel, 2014)—shows that across the Americas, higher socioeconomic status is often classified as White. In the U.S., studies have found that some individuals are more likely to be classified (by interviewers) and identify as White over time as their social position increases, and as Black following prolonged periods of status loss (Saperstein and Penner, 2012).

Conversely, in Latin America and the Caribbean, research has shown that higher status does not consistently “Whiten” racial identification. In fact, scholars have identified varying trends: “polarization” (higher status individuals identifying more frequently as either Black or White); “darkening” (higher status individuals selecting darker racial categories); and “mestizoization” (a shift toward lighter/mixed-race/mestizo classification) (Kelly, 2025; McNamee, 2020; Roth et al., 2022; Telles and Flores, 2013; Telles and Paschel, 2014).

Moreover, research has shown that skin shade does not neatly map onto racial identification in all contexts (e.g., Telles and Paschel, 2014). Taken together, this body of work demonstrates the greater variability and complexity of racialization processes and understandings across the region.

The context of Trinidad and Tobago therefore proves useful for examining the centrality of embodied cues of typicality, namely skin shade, and how these cues differentially shape the socioeconomic capital associated with skin shade (i.e., colorism). Given the complexity of the country’s racial formation, post-independence shifts in the intersection of race, color, and class (Ryan and Stewart, 1995; Tate, 2007), centering stratification based on skin shade in the Trinidadian context may demonstrate how reliance on official classifications, while locally specific, function in similar ways across settings to obscure more nuanced and comprehensive dimensions of inequality.

### 1.2 Colorism in Trinidad and Tobago

Once a British colony, Trinidad and Tobago was shaped by an extractive plantation economy. Prior to British rule, the islands were controlled at various times by colonial powers including the Spanish, French, Dutch, and Courlanders. British authority was formally established in 1814, and in 1889, the unification of Trinidad and Tobago created a single British crown colony (Khadan and Ruprah, 2024; Williams, 1962). Historically, society was structured around a color-based hierarchy: white people at the top, Browns or “coloreds” (often mixed-race individuals) in the middle, and Black people at the bottom. This hierarchy was closely tied to class: Lighter skin was associated with privilege, wealth, and power, while darker skin correlated with poverty and marginalization (Braithwaite, 1953; McCree, 2015; Nettleford, 1998). The arrival of large numbers of (East) Indian laborers beginning in 1839 (Persaud, 2024), alongside imported workers from China, West Africa, and Madeira (i.e., Portuguese), made the country’s ethnoracial composition more complex (Braithwaite, 1953).

British policies exacerbated division between Black and East Indian populations. For example, policies allowed East Indian laborers to acquire land instead of return passage, facilitating a transition to peasant farming, enabling the group to remain in the agricultural sector (Munasinghe, 2018; Reddy, 2011). By 1902, East Indians produced over half the island’s sugar cane and had expanded into cocoa and rice farming. Many emancipated Africans, however, were forced to resort to squatting due to the inflated prices of land bordering estates (Williams, 1962; Kelly, 2023). Despite earlier unity between the two ethnoracial groups during the 1930s labor movements, politics soon became ethnically polarized, with low voter turnout and fragmented ethnically divided support in the 1946 elections (Khadan and Ruprah, 2024).

In 1955, Dr. Eric Williams founded the People’s National Movement, which, although officially multiethnic, was largely aligned with the interests of the Black Trinbagonians. Another salient expression of this inter-ethnoracial tension is persistent differential ethnoracial support for governmental parties (Bahadoorsingh, 1968; Brereton, 2010; Ryan et al., 1979; Stone, 1972). Contemporarily, it has been observed that this ethnoracial political divide is rife with race-baiting among political leaders (Rampersad, 2022), spurring public discourse on the seeming inability of political leaders to transcend these ethnopolitical cleavages (Anon, 2022; Rakhal-Fraser, 2022a, 2022b). However, scholars tend to frame these ethnopolitical tensions as superficial, because ethnoracial conflict or violence has not erupted with much frequency or potency within the country (Reddy, 2011; Sriskandarajah, 2005).

Leading up to independence in 1962, Afro-Trinbagonians were largely urban and educated, Indo-Trinbagonians were rural and land-owning, and White people dominated capital—reflecting a broader social hierarchy that also included the Chinese, Syrians/Lebanese, and Portuguese (Kelly, 2023; Rajkumar, 2006; Reddy, 2011). The intersection of race, color, and class began to shift post-independence due to education reform, economic expansion, and the Black Power movement in the late 1960s and early 1970s (Ryan and Stewart, 1995; Tate, 2007). These changes enabled upward mobility for both Black and East Indian individuals, reshaping the middle class (Ryan, 1991; Schimanski et al., 2018). However, inequality persists, with both groups continuing to trail White people and other smaller ethnoracial groups, particularly in income and education (Kelly, 2023).

Previous studies on colorism across racialized groups have reported consistent findings. For example, in my previous work (Kelly, 2022), I used data from the AmericasBarometer and national censuses, examined ethnoracial inequality in Jamaica and Trinidad and Tobago. I situated the Anglophone Caribbean region’s tripolar racialized framework within broader comparative discussions of race in the Americas. Employing a multidimensional approach to consider both ethnoracial identification and skin shade, I analyzed their effects on three indicators of socioeconomic status. In Trinidad and Tobago, though both racial identity and skin shade were found to be equally effective in explaining observed patterns of inequality, findings indicated that darker-skinned Trinbagonians faced greater disadvantages in education, income, and household amenities.

Adopting a similar multidimensional measure of race, Painter et al. (2019) also found skin shade and asset ownership to be significantly associated: darker skinned respondents were less likely to own homes or a car. Khadan and Ruprah (2024) also find comparable outcomes for income across ethnoracial groups, although their analysis focuses exclusively on Black, East Indian, and mixed-race Trinbagonians.

In addition to how colorism operates across racialized groups in Trinidad and Tobago, it also manifests within-groups. Though there is a dearth of research on the matter, the few existing studies highlight that lighter-skinned Black individuals are often more likely to attain higher status than their darker-skinned counterparts (Bernard, 2015; Doubeni, 2017; Elwin, 2022; Stewart, 2004). Among East Indians, colorism is similarly shaped by caste-influenced norms, with lighter skin shade being highly valued (Braithwaite, 1953; Khan, 2009; Vijaya, 2019), especially in endogamous marriages (Dhillon-Jamerson, 2019).

Skin color should therefore be understood as a salient marker of social status, one that can operate independently of broader racialized categorizations. It thus requires explicit attention in analyses of racialized inequality.

## 2 Data and methods

### 2.1 Data

This study is distinct in offering a quantitative analysis of skin shade stratification by examining the multidimensionality of colorism within a single national population: Trinidad and Tobago. It draws on all available waves of the AmericasBarometer social surveys conducted there: 2010, 2012, 2014, and 2023. The data were collected as part of the Latin American Public Opinion Project (LAPOP), housed at Vanderbilt University, with fieldwork support from the University of the West Indies, St. Augustine. Face-to-face surveys were administered in English to nationally representative samples of voting-age adults. The complex sampling design used stratified, multi-stage cluster sampling based on three factors: (1) municipality size, (2) urban versus rural classification, and (3) regions. This approach produced an estimated sampling error of approximately 2.4–2.5% across waves and yielded sample sizes ranging from 1,505 to 4,207 respondents. The four waves were merged into a combined unweighted sample of 8,878 individuals.

### 2.2 Measures

Socioeconomic wellbeing was measured by two key indicators: educational attainment and household amenities.

In the 2010, 2012, and 2014 waves, education was measured as a continuous variable ranging from 0 to 18 years of schooling. In 2023, it was measured as a seven-category ordinal variable. For consistency across survey years, educational attainment was recoded into four categories: 0 = no formal education (none), 1 = primary-level education (1–7 years of schooling), 2 = secondary-level education (8–12 years; reference), and 3 = tertiary-level education (13 or more years) (Esnard, 2021). In Trinidad and Tobago, some students may choose to return to high school for an additional 2 years of study, after which they take the Caribbean Advanced Proficiency Examination (CAPE) and receive an associate degree (Caribbean Examinations Council, 2025). However, because not all students pursue this option—many proceed directly to college—I classify this level of education within the broader tertiary education category.

Household amenities were measured using a standardized factor score based on a relative wealth index developed by LAPOP (Cordova, 2008). The index included eight consistently available items across all waves: refrigerator, cellular phone, washing machine, microwave, indoor plumbing, computer/laptop, flat panel TV, and internet access. Exploratory factor analysis identified two factors with eigenvalues greater than one; however, only one factor was retained after removing items with factor loadings below a 0.3 (i.e., refrigerator and cellphone; Kaiser-Meyer-Olkin measure of sampling adequacy = 0.690). The remaining 6-items loaded onto a single factor (eigenvalue = 1.655), which was then standardized, with higher number indicating greater household amenities access (Cronbach’s alpha = 0.700) (see Kelly, 2020, 2022, 2024).

Skin color was assessed by interviewers using the Project on Ethnicity and Race in Latin America (PERLA) palette, an ordinal 11-point scale ranging from 1 (lightest) to 11 (darkest). To distinguish between intraracial and interracial colorism, I decompose the measure into two components using group-mean centering: (1) a between-group component representing the average skin color rating for each ethnoracial group and (2) a within-group component, representing the deviation of each respondent from their respective ethnoracial group’s mean skin color. For analyses, I restrict the sample to respondents self-identified as Black, mixed, or (East) Indian. The mixed category includes Douglas (Black–East Indian parentage) and other mixed-race individuals, though the LAPOP data do not disaggregate this group—representing both a limitation and an avenue for further research. This decomposition separates the effects of skin shade stratification that operates within racial groups from those that reflect the broader ethnoracial hierarchy linked to skin shade across groups.

Sociodemographic control variables included sex (1 = female), residential location (reference = urban), age (18–98), marital status (1 = married), employment status (1 = employed), survey year (to control for period effects), and interviewer skin color (measured on a scale from 1 to 11), which was included to control for interviewer effects (see Hill, 2002; Kelly, 2022). Additionally, educational attainment was used as a control variable in models predicting household amenities, given its strong connection with asset ownership. Household income was excluded to retain the largest possible sample. Although colorism may influence covariates, such as marital status and employment, including these variables do not compromise the analyses as the primary aim of the study is to estimate the direct effect of skin shade on socioeconomic outcomes, net of sociodemographic controls.

### 2.3 Methods

I first present weighted summary statistics for all outcome and independent variables along with bivariate distributions of skin color by ethnoracial status. To examine the relationship between skin color and socioeconomic wellbeing, I employed multilevel mixed effects models. As respondents were nested within primary sampling units (PSUs), this modeling strategy accounted for the stratified and clustered sampling design of the AmericasBaromter, while allowing for the estimation of both within-group (intraracial) and between-group (interracial) racialized skin shade differences. Specifically, I estimated the association between skin color and educational attainment using multilevel ordered logit models and used multilevel linear regression models to assess skin color differences in household amenities.

To confirm the appropriateness of multilevel modeling, I first estimated a null model and calculated the intraclass correlation coefficient (ICC) and the likelihood ratio test. I then examined intra- and interracial differences through stepwise modeling, employing a total of three models. Model 1 assessed intraracial differences (i.e., within-group skin color [whether respondents are darker or lighter relative to their racial group’s mean skin color]). Model 2 added between-group skin color (mean skin color for each racial group) to Model 1. Model 3 incorporated interactions of within-group skin color and racial group to evaluate whether the effects of intraracial colorism varied across the three ethnoracial groups.

All models included random intercepts (without random slopes), Level 1 sociodemographic controls, survey year, and sample weights. Robust standard errors were used to adjust for heteroskedasticity and remaining survey design complexities. To compare model fit across nested specifications, the Akaike Information Criterion (AIC) are reported, and likelihood-ratios were computed from re-estimated models without robust standard errors (not shown). In models predicting educational attainment and household amenities, I excluded the category “no formal education” due to the small sample size.

To assess whether the effects of skin shade stratification varied across ethnoracial groups, I first conducted a joint significance test for Model 3. I then estimated predicted marginal effects from this model to quantify the expected change in educational attainment or household amenities associated with one-unit increase in within-group skin color for each group. Additionally, to illustrate the magnitude of colorism effects for both socioeconomic outcomes—and to compare magnitudes across groups—I graphed predicted values at the lighter-than-average (−2), average (0), and darker-than-average (+2) within-group skin color positions for each ethnoracial group.

## 3 Results

### 3.1 Descriptive findings

Table 1 presents all variables used in the multilevel analyses, detailing their measurement, level, and definitions.

**Table 1:** Variables used in multilevel analysis.

Variables	Level	Measurement/coding	Description
Educational attainment	1	Ordered categorical: 0 = none; 1 = primary; 2 = high school; 3 = tertiary	Respondent’s completed educational attainment
Household amenities	1	Continuous standardized factor score derived from factor analysis of 8 household-amenity items, with the final factor extracted from 6 retained items	Standardized household wealth measure reflecting relative material wellbeing; higher scores = greater wealth
Skin color		Continuous 1–11 PERLA palette (from lightest to darkest)	Interviewer rated skin color
Within-group skin color	1	Continuous deviation score: skin color – between race skin color	Individual’s skin color relative to their racial group mean; values can be negative or positive
Between-group skin color	1	Group mean of skin color within each ethnoracial category	Average skin color of racial group; constant for all individuals in the same race
Ethnoracial Category	1	Categorical: Black, Mixed, East Indian	Cluster variable defining groups with random intercepts
Female	1	Binary: 1 = female, 0 = male	Respondent’s sex
Age (in years)	1	Continuous (18–98)	Respondent’s age at survey
Employed	1	Binary: 1 = employed, 0 = not employed	Current employment status
Married	1	Binary: 1 = married, 0 = unmarried	Legal marital status
Urban residency	1	Binary: 1 = urban, 0 = rural	Indicator for living in an urban area
Interviewer skin color	1	Continuous (1–11)	Interviewer’s rated skin color based on the PERLA color scale
Survey year	1	Dummy indicators: 2010 (ref), 2012, 2014, 2023	Year of data collection
Primary Sampling Unit (PSU)	2	Categorical cluster identifier	Survey sampling cluster; modeled with a random intercept

Table 2 presents weighted summary statistics for the pooled sample. Respondents were equally distributed by gender and had an average age of 39.7 years. Sixty percent were employed, 76.6% resided in urban areas, and approximately one-third were married. Educational attainment was relatively high: 54.2% had completed or had some level of secondary education and 29.6% had some level of tertiary education. The household amenities standardized factor score had a mean of −0.009, indicating that respondents in the sample had average access.

**Table 2:** Weighted summary statistics for key variables used in multilevel analyses (*N* = 8,157).

Variables	Mean	S.E.	Min	Max
Educational attainment	–	–	1	3
Primary	0.162	0.005	–	–
High school	0.542	0.007	–	–
Tertiary schooling	0.296	0.007	–	–
Household amenities	−0.009	0.009	−2.585	0.632
Skin color	6.434	0.024	1	11
Within-group skin color	0.104	0.022	−5.201	5.354
Between-group skin color	6.330	0.010	5.646	7.201
Black (group mean skin color)	7.201	–	–	–
Mixed (group mean skin color)	5.646	–	–	–
East Indian (group mean skin color)	5.755	–	–	–
Ethnoracial category	–	–	1	3
Black	0.418	0.007	–	–
Mixed	0.264	0.006	–	–
East Indian	0.318	0.007	–	–
Female	0.499	0.007	0	1
Age (in years)	39.710	0.224	18	98
Employed	0.602	0.007	0	1
Married	0.329	0.007	0	1
Urban residency	0.766	0.006	0	1
Interviewer skin color	6.077	0.023	1	11
Survey year	–	–	2010	2023
2010	0.172	0.005	–	–
2012	0.172	0.005	–	–
2014	0.477	0.007	–	–
2023	0.179	0.005	–	–
PSU	898.719	24.1001	1	9,705

Means and standard errors (S.E.) are rounded to three decimal places.

Respondents with no formal schooling (*n* = 49) were excluded due to small cell size.

The average skin color rating across the pooled sample was 6.4 (on an 11-point scale), with respondents identifying as Black (42%), mixed-race (26%), and East Indian (32%). The within-group skin color variable—which captures the deviation of each respondent’s skin shade from their intraracial group’s mean skin color—displayed wide variation, ranging from −5.201 to 5.354. The between-group skin color variable defined as the average skin color rating within each ethnoracial group, consisted of mean skin color ratings of 7.201, 5.646, and 5.755 for Black, mixed, and East Indian respondents, respectively.

**Figure 1:**
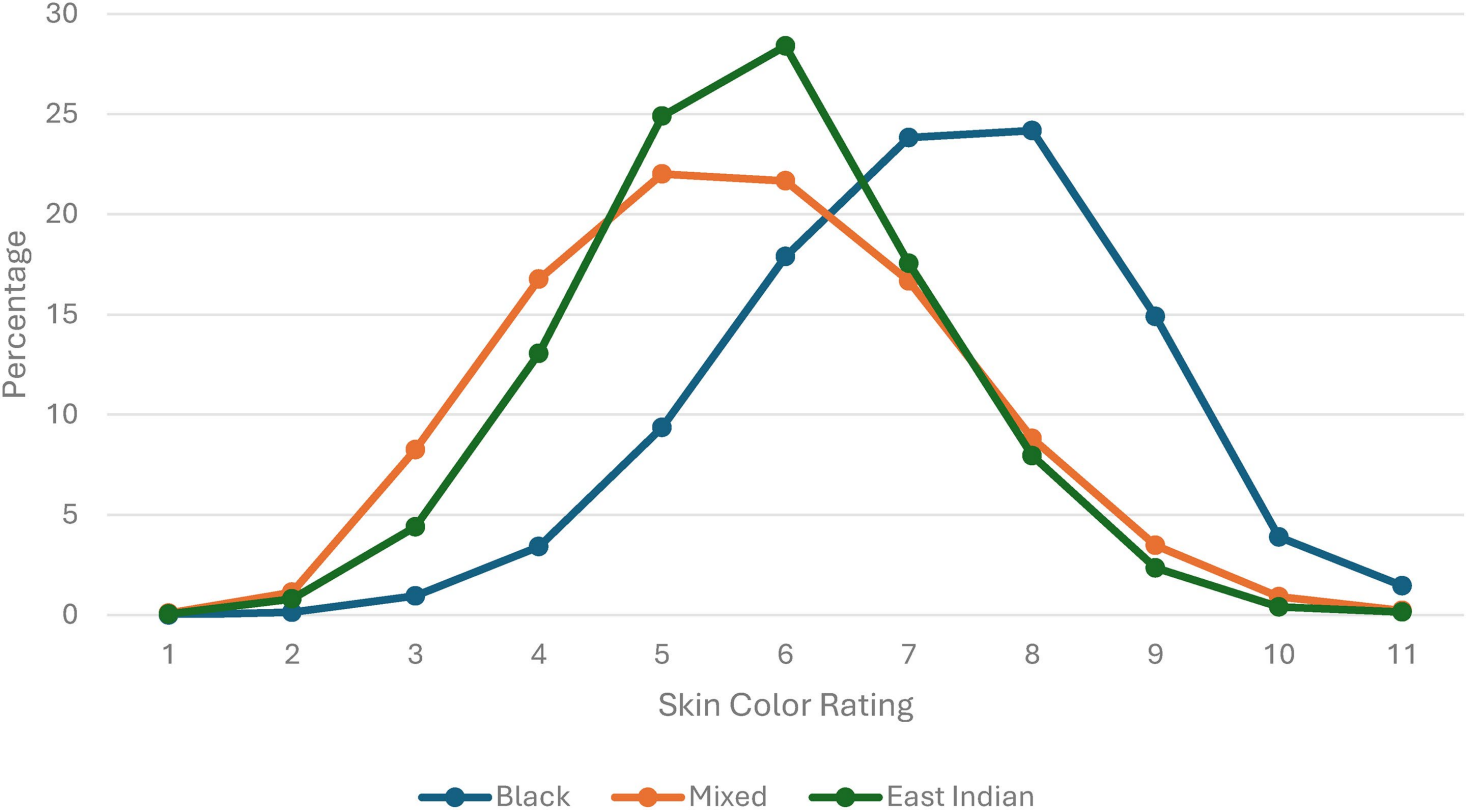
Skin color distribution within ethnoracial groups for pooled sample.

Figure 1 shows the distribution of interviewer-rated skin color for Black, mixed, and East Indian self-identified respondents on an 11-point scale. Although all three groups are represented across the full range of the scale, Afro-Trinbagonians are more heavily represented at the darker end, particularly between 7 (23.8%) and 8 (24.2%). Mixed respondents display the lightest overall distribution, clustering between ratings 4 and 7 (77.1%). Indo-Trinbagonian respondents are most concentrated at ratings 5 (24.9%) and 6 (28.4%), with percentages declining sharply thereafter. All three ethnoracial groups show relatively low representation at the extreme ends of the scale (1–2 and 10–11). These patterns highlight the heterogeneity of skin shade both within- and between-groups.

### 3.2 Regression analyses: educational attainment

Table 3 presents weighted coefficients from four multilevel mixed-effects ordered logit models predicting educational attainment. The null model provides the baseline distribution of educational attainment across PSUs. Model 1 introduces the within-group component of skin color (skin_within; deviation from group skin shade average), Model 2 adds the between-group component (skin_mean; average skin shade of racial group), and Model 3 includes interactions between within-group skin color and ethnoracial category. Likelihood ratio test of the null model reveal that multilevel modeling was a better fit for the data than a standard ordered logit model (*χ*^2^ = 313.35, *p* < 0.001).^2^ This finding justifies the use of multilevel modeling when examining educational difference among the sample.

**Table 3:** Weighted coefficients from mixed-effects ordered logit regression predicting educational attainment (*N* = 8,159).

Variables	Null model	Model 1	Model 2	Model 3
Within-group skin color (skin_within)	–	−0.145*** (0.019)	−0.147*** (0.019)	−0.098*** (0.023)
Between-group skin color (skin_mean)	–	–	−0.146*** (0.040)	−0.151*** (0.040)
Interaction: race × skin_within				
Mixed × skin_within	–	–	–	−0.065 (0.041)
East Indian × skin_within	–	–	–	−0.119** (0.047)
Female	–	0.048 (0.065)	0.044 (0.065)	0.040 (0.066)
Age	–	−0.036*** (0.002)	−0.036*** (0.002)	−0.036*** (0.002)
Employed	–	0.462*** (0.064)	0.470*** (0.064)	0.469*** (0.064)
Married	–	0.402*** (0.072)	0.379*** (0.081)	0.375*** (0.073)
Urban	–	0.113 (0.080)	0.091 (0.081)	0.095 (0.081)
Interviewer skin color	–	−0.034 (0.020)	−0.031 (0.020)	−0.031 (0.020)
Survey year (ref = 2010)				
2012	–	0.448*** (0.125)	0.469*** (0.125)	0.462*** (0.126)
2014	–	0.936*** (0.119)	0.960*** (0.121)	0.966** (0.121)
2023	–	0.816*** (0.118)	0.844*** (0.118)	0.843*** (0.118)
Random intercept variance	0.383 (0.055)	0.284 (0.045)	0.279 (0.045)	0.277 (0.044)
ICC	0.104 (0.013)	0.080 (0.012)	0.078 (0.012)	0.078 (0.012)
AIC	15,938.71	15,026.86	15,007.17	**14,999.50**

Source: 2010–23 AmericasBarometer social survey for Trinidad and Tobago.

Coefficients and robust standard errors (in parentheses) are rounded to three decimals and weighted. Standard errors in Models 1–3 are robust and clustered by primary sampling units (PSUs). Skin color is decomposed into within-race and between-race components, with higher values indicating darker skin shades. Respondents are nested within 498 PSUs. ICC values represent the proportion of variance in the latent outcome attributed to PSUs. The analytic sample excludes respondents with no formal schooling (*n* = 49). Model 0 is the unconditional maximum-likelihood model, and all models are mixed-effects ordered logit models with random intercepts for PSUs. The model with the lowest AIC statistic (highlighted in bold) represents the best overall fit.

*p < 0.05. **p < 0.01. ***p < 0.001.

Model 1 of Table 3 estimates the association between within-group skin color and educational attainment, controlling for sociodemographic characteristics and survey year. Results reveal that the two measures are significantly associated. Specifically, results show that respondents with darker skin, relative to intra-group members, have significantly lower odds of completing higher levels of education (*β* = −0.145, *p* < 0.001).

Model 2 of Table 3 incorporates both within-group skin color and between-group skin color to examine the intraracial and interracial impacts of skin shade. Findings show that both components of skin color are statistically significant. For intraracial differences, darker skin shade continues to be negatively and significantly associated with higher levels of education (*p* < 0.001). This is also the same for interracial skin shade differences; respondents from darker-skinned ethnoracial groups, on average, experience further educational disadvantage. That is, these respondents have a reduced odds of reaching higher education categories, even after accounting for their individual skin shade (*β* = −0.146, *p* < 0.001).

According to Model 3, which includes interactions between within-group skin color and ethnoracial group, the effects of skin shade vary significantly across groups. Among Black respondents, within-group skin color decreases but remains negative and statistically significant (*β* = −0.098, *p* < 0.001). The interaction of within-group skin color and the mixed ethnoracial category is negative but not statistically significant, whereas results from interactions for within-group skin color and East Indian respondents are both negative and significant (*β* = −0.119, *p* < 0.01). This suggests that the negative and significant association between skin shade and educational attainment is stronger for East Indian respondents compared to Black respondents (reference group). A joint Wald test confirms that the within-group skin-color slopes differ significantly across the three ethnoracial groups (*χ*^2^(2) = 7.17, *p* < 0.05), providing strong evidence that the effect of within-group skin color on educational attainment is not uniform across groups.

#### 3.2.1 Marginal effects of within-group skin color on educational attainment

To further explicate differences in the effect of intraracial colorism across ethnoracial groups, Table 4 presents average marginal effects (AMEs) estimating how within-group variation in skin shade affects the predicted probability of attaining tertiary education across ethnoracial groups (based on Model 3). Across all groups, darker skin shade is significantly associated with a lower likelihood of attaining tertiary level education. A one-unit increase in within-group skin color darkness is associated with a 1.7, 2.9, and 3.8% decrease in the probability of gaining tertiary education among Afro-, mixed, and Indo-Trinbagonians, respectively (*p* < 0.001). Despite this general trend in the negative impact of darker skin on educational outcome, pairwise comparison reveal that this disparity is only statistically significant for the comparison between Black and East Indian respondents (difference = −0.021, *p* < 0.001).

**Table 4:** Average marginal effects of within-group skin color on the probability of attaining tertiary education, by ethnoracial group (*N* = 8,159).

Average marginal effects (AME) by ethnoracial group		
Ethnoracial group	AME (dy/dx)	S.E.
Black	−0.017***	0.004
Mixed	−0.029***	0.006
East Indian	−0.038***	0.007

Pairwise comparisons of AMEs		
Comparison	Difference in AME	S.E.
Mixed vs. Black	−0.011	0.007
East Indian vs. Black	−0.021***	0.008
East Indian vs. Mixed	−0.009	0.009

AMEs estimated using margins with predictive margins for outcome category 3 (tertiary education). Standard errors are robust and clustered by PSUs. Values are rounded to three decimals. Predicted probabilities are based on Model 3 (mixed-effects ordered logit with interactions between within-group skin color and race).

*p < 0.05. **p < 0.01. ***p < 0.001.

**Figure 2:**
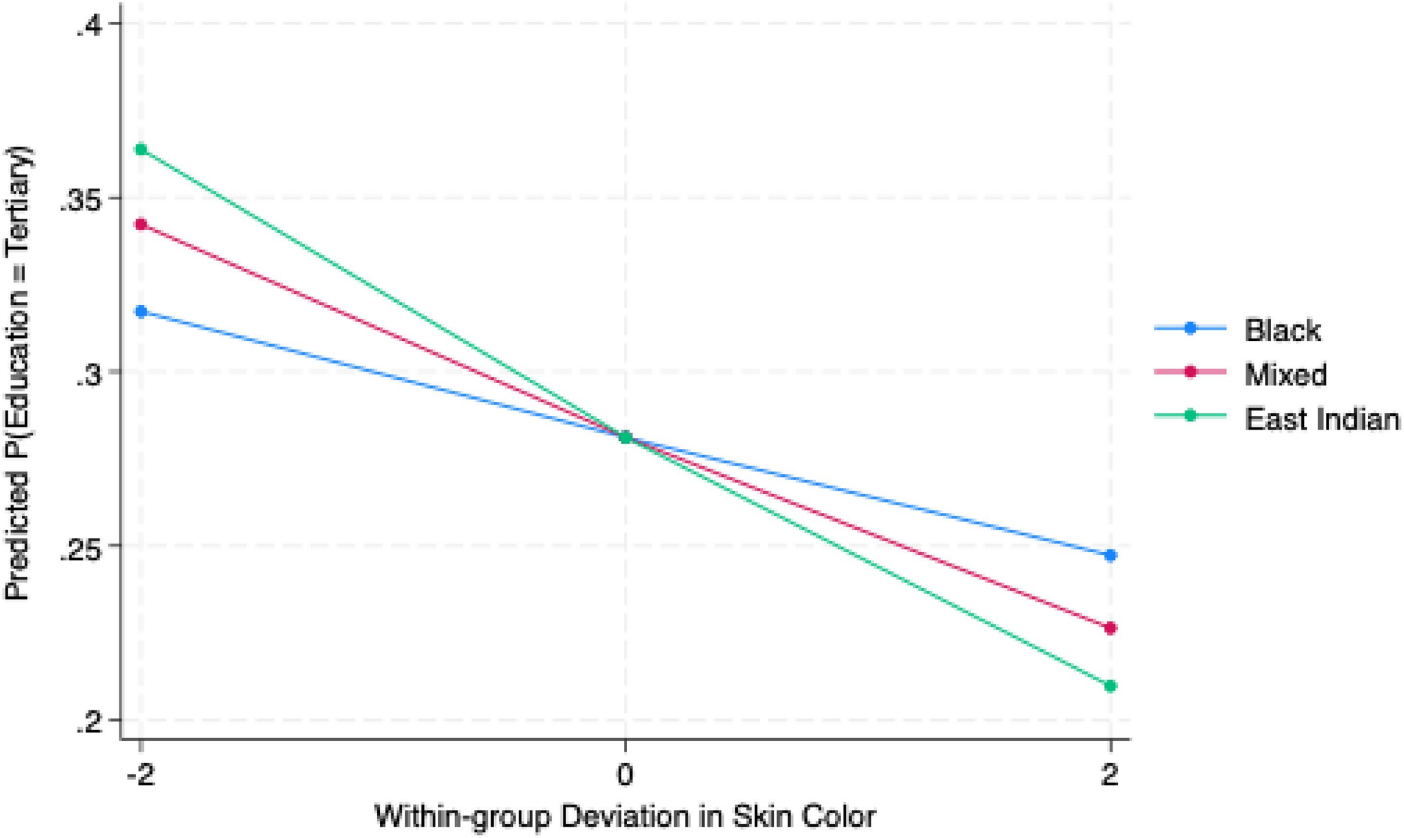
Predicted probability of tertiary education by within-group skin color deviation and ethnoracial group. Predicted probabilities are based on Model 3 (mixed-effects ordered logit regression with random intercepts for PSUs). Skin-color deviation represents within-group differences from the ethnoracial group mean (−2 = lighter than group average; 0 = group average; +2 = darker than group average). Confidence intervals are suppressed for clarity. Estimates use sampling weights and robust standard errors clustered by PSUs.

Figure 2 plots predicted probabilities of tertiary-level educational attainment for three within-group skin color deviation scores: −2 (lighter-than-average), 0 (average), and +2 (darker-than-average). Lighter-than-average East Indian respondents report the highest probability of attaining tertiary education, followed by mixed and Black respondents, respectively. However, conversely, darker-than-average Indo-Trinbagonian respondents have the lowest probability of reaching tertiary education. In fact, the group’s predicted probability decreases by 15% points from lighter-than-average to darker-than-average skin colors. Therefore, the figure illustrates that for tertiary-level education, the strongest effects of intraracial colorism appears among East Indian respondents, followed by mixed-race individuals, and Black respondents.

### 3.3 Household amenities

Table 5 presents weighted coefficients from four multilevel mixed-effects linear regression models predicting household amenities. Likelihood ratio test of the null model reveals that multilevel modeling was a better fit for the data than a standard ordered logistic regression model (*χ*^2^ = 279.08, *p* < 0.001) (see text footnote 2); justifying multilevel modeling.

**Table 5:** Weighted coefficients from multilevel mixed-effects linear regression predicting household amenities (*N* = 8,159).

Variables	Null model	Model 1	Model 2	Model 3
Within-group skin color (skin_within)	–	−0.046*** (0.005)	−0.047*** (0.005)	−0.033*** (0.008)
Between-group skin color (skin_mean)	–	–	−0.021* (0.010)	−0.022* (0.020)
Interaction: race × skin_within				
Mixed × skin_within	–	–	–	−0.030** (0.012)
East Indian × skin_within	–	–	–	−0.014 (0.012)
Female	–	0.017 (0.016)	0.016 (0.016)	0.017 (0.016)
Age	–	−0.004*** (0.001)	−0.004*** (0.001)	−0.004*** (0.001)
Employed	–	0.067*** (0.019)	0.068*** (0.019)	0.067*** (0.019)
Married	–	0.203*** (0.019)	0.200*** (0.018)	0.200*** (0.018)
Urban	–	0.070** (0.027)	0.067** (0.027)	0.068** (0.027)
Interviewer skin color	–	−0.017*** (0.005)	−0.017*** (0.005)	−0.017*** (0.005)
Survey year (ref = 2010)				
2012	–	0.033 (0.028)	0.035 (0.028)	0.034 (0.028)
2014	–	0.174*** (0.028)	0.177*** (0.028)	0.179*** (0.028)
2023	–	0.350*** (0.026)	0.353*** (0.026)	0.355*** (0.026)
Education (ref = primary)				
High school	–	0.265*** (0.032)	0.265*** (0.032)	0.265*** (0.032)
Tertiary	–	0.551*** (0.031)	0.550*** (0.031)	0.548*** (0.031)
Intercept	−0.045*** (0.013)	−0.384*** (0.059)	−0.255*** (0.090)	0.258 (0.138)
Random effects				
PSU variance	0.036 (0.005)	0.014 (0.002)	0.013 (0.002)	0.013 (0.002)
Residual variance	0.383 (0.013)	0.315 (0.011)	0.314 (0.011)	0.314 (0.011)
ICC	0.086 (0.013)	0.041 (0.007)	0.041 (0.007)	0.041 (0.007)
AIC	16,062.95	14,111.69	14,108.06	**14,101.78**

Source: 2010–23 AmericasBarometer social survey for Trinidad and Tobago.

Household amenities scores are derived from a single standardized factor. Coefficients and robust standard errors (in parentheses) are rounded to three decimals. Models 1–3 use sampling weights and robust standard errors clustered by primary sampling units (PSU). Respondents are nested within 498 PSUs, and all models include PSU-level random intercepts. Skin color is decomposed into within-race and between-race components, coded from lightest to darkest. The analytic sample excludes respondents with no formal schooling (*n* = 49). Model 0 is the unconditional model. All models are weighted mixed-effects linear regressions. The model with the lowest AIC statistic (highlighted in bold) represents the best overall fit.

*p < 0.05. **p < 0.01. ***p < 0.001.

Consistent with findings on educational attainment, the results show that having darker skin relative to one’s group mean is negatively and significantly associated with fewer household amenities (*β* = −0.046, *p* < 0.001). After including between-group skin color in Model 2, the coefficient for within-group skin color remains statistically significant (*p* < 0.001). Results for between-group skin color also show that respondents of racially darker groups have fewer household amenities (*β* = −0.021, *p* < 0.05), independent of within-group skin color. Although statistically significant, the effect size is comparatively small.

Model 3 present the results for household amenities after including interactions between race and within-group skin color to test the intraracial effects of colorism across ethnoracial categories. These interactions show that, compared to Black respondents, the penalty of having darker skin is significantly greater for mixed-race respondents (*β* = −0.030, *p* < 0.01). For East Indian respondents, this interaction is negative but not statistically significant. A Wald test confirms that the interaction coefficients jointly differ from the reference group (Afro-Trinbagonians) (*χ*^2^(2) = 6.46, *p* < 0.05).

#### 3.3.1 Marginal effects of within-group skin color on household amenities

AMEs presented in Table 6 show that darker skin relative to the ethnoracial group mean is associated with significantly fewer household amenities across all groups. Each unit increase in the darkness of skin color has an associated 0.033-unit, 0.064-unit, and 0.048-unti decrease in household amenities for Black, mixed, and East Indians Trinbagonians, respectively (*p* < 0.001). The intraracial penalty of darker skin is therefore strongest among mixed respondents, followed by Indo- and Afro-Trinbagonians. Pairwise comparisons confirm findings in Table 5 as only comparison between Black and mixed respondents are statistically significant (difference = −0.030, *p* < 0.01).

**Table 6:** Average marginal effects of within-group skin color on household amenities by ethnoracial group (*N* = 8,159).

Average marginal effects (AME) by ethnoracial group		
Ethnoracial group	AME (dy/dx)	S. E.
Black	−0.033***	0.008
Mixed	−0.064***	0.010
East Indian	−0.048***	0.010

Pairwise comparisons of AMEs		
Comparison	Difference in AME	S.E.
Mixed vs. Black	−0.030**	0.012
East Indian vs. Black	−0.014	0.012
East Indian vs. Mixed	−0.016	0.014

AMEs estimated using margins based on Model 3. Standard errors are robust and clustered by PSUs. Values are rounded to three decimals. The analytic sample excludes respondents with no formal schooling (*n* = 49).

*p < 0.05. **p < 0.01. ***p < 0.001.

**Figure 3:**
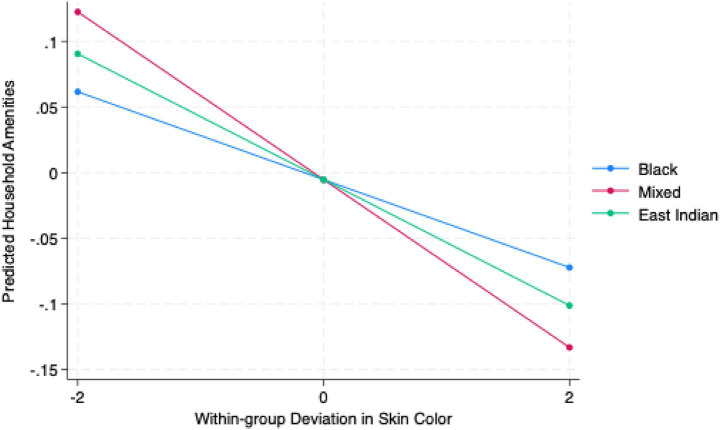
Predicted values of household amenities by within-group skin color deviation and ethnoracial group. Predicted probabilities are based on Model 3 (mixed-effects linear regression with random intercepts for PSUs). Skin-color deviation represents within-group differences from the ethnoracial group mean (−2 = lighter than group average; 0 = group average; +2 = darker than group average). Confidence intervals are suppressed for clarity. Estimates use sampling weights and robust standard errors clustered by PSUs.

Figure 3 presents predicted values for household amenities by within-group skin color deviations across the three ethnoracial groups. Lighter-than-average (−2) mixed-race respondents have the greatest predicted household amenities, followed by East Indian and Black respondents. These patterns reverse for darker-than-average (+2) individuals in each ethnoracial group. Mixed respondents experience a 0.11-unit decrease in predicted household amenities from lighter-than-average to darker-than average skin colors, with Indo- and Afro-Trinbagonians following with smaller declines, respectively. These patterns illustrate that intraracial colorism decreases access to household amenities for all ethnoracial groups, with the strongest effects observed among mixed-race individuals.

### 3.4 Model fit

To assess model fit, a series of likelihood-ratio tests were conducted to compare each of the three models presented in Tables 3, 5. For educational attainment, likelihood-ratio tests show that colorism functions at multiple levels and varies across the three ethnoracial groups. Including the within-group skin color measure in the null model significantly improved model fit (*χ*^2^(10) = 971.95, *p* < 0.001), demonstrating that intraracial differences in skin shade are associated with different educational outcomes. Likewise, likelihood-ratio tests comparing Model 1 to Model 2 (*χ*^2^(1) = 34.62, *p* < 0.001) and Model 2 to Model 3 (*χ*^2^(2) = 12.64, *p* < 0.01) show that adding between-group skin color improves model fit and that within-group skin color effects vary across ethnoracial groups, respectively. Likelihood-ratio tests for household amenities yield a similar pattern, indicating that each stepwise progression significantly improved fit (*p* < 0.05). This pattern is consistent with the AIC statistics, which decrease across models, indicating better overall fit. Model 3 is thus the best overall fit for educational attainment (AIC = 14,999.50) and household amenities (AIC = 14,101.78).

## 4 Discussion

This study extends the literature on skin-shade stratification by centering Trinidad and Tobago as a case for examining how skin tone structures socioeconomic wellbeing both inter- and intraracially. The results reveal a persistent association between skin shade and socioeconomic outcomes: respondents with darker skin had lower odds of attaining higher levels of education and possessed fewer household amenities. Notably, because education and employment were controlled for in the analysis of household amenities, the significant disadvantages associated with darker skin suggest that additional mechanisms—beyond formal qualifications—may mediate these outcomes. These findings align with prior studies in Trinidad and Tobago (e.g., Kelly, 2022; Painter et al., 2019) and contribute to a broader body of research documenting global pigmentocracies. In doing so, the study enhances our understanding of how skin shade functions as a distinct axis of inequality (e.g., Monk, 2016; Telles, 2014; Telles et al., 2025).

The analysis then assessed intraracial variation by skin shade and evaluated the relative effects of colorism across ethnoracial groups: Black, mixed, and East Indian. Results again showed that differences in skin color stratified access to education and household amenities. Among Afro- and mixed-Trinbagonian respondents, these disparities align with longstanding patterns in which lighter skin is associated with perceived respectability, competence, and social mobility (Braithwaite, 1953; McCree, 2015; Nettleford, 1998; Stewart, 2004; Tate, 2007). Moreover, findings are consistent with previous research that has documented significant disparities by skin shade within various racial groups (e.g., Hall, 2022; Hall and Mishra, 2024; Hunter, 2007; Kelly, 2024; Monk, 2015, 2021b; Monk et al., 2021; Ryabov, 2016).

The most pronounced disparities in educational access were observed among Indo- Trinbagonians, suggesting that colorism is deeply entrenched within the community, where lighter skin is valorized through cultural norms and matrimonial preferences (Braithwaite, 1953; Dhillon-Jamerson, 2019; Khan, 2009; Vijaya, 2019). As Khan (2009, p. 100) notes, although East Indians are ideologically distanced from “Trinidadian nationalism (Afro + Euro = authentic, Creole Trinidadian) [see also Brereton, 2010; Munasinghe, 2019]; … they are not shorn of the meaning of, nor outside the consequences of, color and race.” This indicates that, despite Indo-Trinbagonians’ marginalization from the dominant national identity, they remain entangled in the country’s ethnoracial and pigmentocratic hierarchies. Historical systems of stratification persist, adapt, and continue to shape community dynamics and mobility, even within diasporic contexts with longstanding histories outside the Indian subcontinent.

Additionally, mixed respondents experience the strongest impact of intraracial colorism on access to household amenities. This finding suggests that phenotype, beyond skin shade, serves as a particularly powerful marker of social class (Hordge-Freeman, 2015; Monk, 2022; Wade, 2012) within this group. Because ancestry was not specified for the “mixed” category in the LAPOP data, it is likely that skin shade intersects with other phenotypic traits—such as hair texture, facial features, or body type—to shape social privilege. This complicates the often-assumed advantage of mixed-race identification in Caribbean societies, highlights internal stratification within ostensibly “privileged” intermediary ethnoracial groups, and suggests that structural inequalities are shaped by a wider range of physical cues (Hunter, 2011, 2021; Monk, 2022; Wade, 2012). The category of “mixed” is deeply heterogeneous, reflecting wide variation in appearance, ancestry, and generational legacy (e.g., Dougla vs. other mixed ancestry). This diversity complicates assumptions of racial typicality and underscores the limits of nominal categories in capturing lived social stratification. In this respect, findings from the study extend and affirm Monk’s (2022) argument that multiple-race identification disrupts conventional boundaries and calls for reconsideration of “how differences relate to inequality beyond census categories” (p. 20).

While findings on the consistent and significant predictive power of skin shade on outcomes are not surprising, this study is novel in that it uncovers unexpected patterns in the magnitude of intraracial colorism effects across ethnoracial groups. As some U.S.-based research illustrates that within-group skin shade differences among Black Americans can produce inequalities greater than those between racial groups (Keith and Herring, 1991; Monk, 2015, 2022), one might have expected the penalty for darker skin shade to be most severe among Black respondents. However, the results from this study reveal the opposite pattern.

These findings may be explained by ethnoracial prototypicality and the racialization of skin shade. Darker skin is central to how Blackness is constructed and stereotyped (Eberhardt et al., 2006; Maddox and Gray, 2002), therefore, additional darkening may do little to alter how Black respondents are perceived. In contrast, for East Indian and many mixed respondents—groups generally racialized as lighter than Black people—darker skin shade constitutes a clearer deviation from group-based prototypicality, which may lead to stronger penalties. As shown in Figure 1, darker-skinned East Indian and mixed individuals deviate more noticeably from their group’s prototypical molds, often imagined as lighter. Because they stand farther from this perceived typicality, they may face greater bias or misclassification (e.g., Maddox et al., 2022).

Moreover, skin shade can be understood as a form of embodied cultural capital, whose value depends on the social and ethnoracial context in which it is interpreted (Ellis and Destine, 2023; Monk, 2016; Telles et al., 2025). Although lighter skin often carries social advantages, the size of the benefit—or penalty—varies across groups. For East Indian and mixed respondents, who are generally associated with lighter skin, darker skin shade may sharply reduce the value of this embodied capital. For Black respondents, however, the relative impact may be smaller because evaluations of Blackness already assume darker skin. As a result, the meaning and consequences of skin shade differ across ethnoracial groups, contributing to the uneven patterns of intraracial colorism observed in this study.

Furthermore, despite longstanding ethnoracial tensions in Trinidad and Tobago between Afro- and Indo-Trinbagonians, Blackness may not be as heavily structurally disadvantaged. Post-emancipation social and economic policies, particularly those pursued by the country’s first prime minister Dr. Eric Williams, contributed substantially to the country and Afro-Trinbagonian economic advancement (Reddy, 2011; Sriskandarajah, 2005; Stone, 1972). In my own work (Kelly, 2023), using data from five national censuses (1970–2011), I found that East Indian respondents were consistently and significantly disadvantaged in education and personal income compared to Black respondents. Building on these findings, the present study further disaggregates outcomes to identify who within these groups is most disadvantaged—suggesting that it may be specifically darker-skinned Indo-Trinbagonians who face disproportionate educational barriers.

These findings contradict US-dominant narratives of Asian exceptionalism or the “model minority” myth—which often frame Black disadvantage as singular and Asians as universally privileged (Kim, 1999, 2018). This complicates simplified assumptions about intergroup privilege and instead reveals the layered and context-specific dynamics of colorism in Trinidad and Tobago, aligning with the findings of Kelly (2022). Moreover, the findings indicate that the social meaning of skin color—and its consequences for education and household amenities—is not uniform across ethnoracial groups but shaped by each group’s sociocultural position within the broader national racial order (Khan, 2009; McCree, 2015). Findings thus accentuate the complex and variable nature of pigmentocracies (Telles, 2014) and emphasize the need for more nuanced, comparative analyses of intraracial colorism across heterogeneous populations.

This study contributes to bridging regional and methodological traditions by illustrating how both intraracial and interracial dimensions of colorism shape distinct patterns of inequality. Theoretically, the results of this study further emphasize the need to disaggregate race-based analyses to account for between-group and within-group variation in embodied cues, such as skin shade. Traditional census categories may obscure substantial heterogeneity in how inequality is experienced and perpetuated and hence limit policies set to address disparities based on group differences. For example, anti-discrimination measures such as the Equal Opportunity Act of 2000 have been introduced in Trinidad and Tobago (Ministry of the Attorney General and Legal Affairs, 2016). As is common with many such policies, it centers on “race” defined as “a group of persons of common ethnic origin, color or of mixed race” (p. 7). The law thus relies on census categories to recognize and address disparities. The evidence presented here demonstrates that disparities by skin shade, both inter- and intraracially, are equally significant and call for greater attention. Moreover, the conflation of race and color in the above anti-discrimination policy further underscores the importance of centering embodied cues when examining and addressing inequalities more holistically.

By bringing the often-overlooked case of Trinidad and Tobago into wider conversations on skin shade stratification, the findings reinforce that skin shade remains a powerful dividing line in Trinidad and Tobago, not only across racialized groups but within them. This echoes broader global patterns of colorism, yet the Trinidadian context adds a distinctive regional perspective.

## Data Availability

Publicly available datasets were analyzed in this study. This data can be found at: https://www.vanderbilt.edu/lapop/raw-data.php.

## Footnotes

1 A point to note here is that in regions such as Asia and North Africa, lighter skin has long functioned as a marker of class and privilege independent of Western racial hierarchies. My aim is not to superimpose the centrality of Eurocentric ideals onto all contexts, but rather to highlight how racialization operates within the Americas—the region of the current study—and the racial logics to which immigrants in this region may be subjected.

2 Likelihood ratio test derived from unweight null model, not shown.

## References

[ref1] Adames A. (2023). The cumulative effects of colorism: race, wealth, and skin tone. Soc. Forces 102, 539–560. doi: 10.1093/sf/soad038

[ref2] Anon. (2022). T&T divided by politics and race. Available online at: https://guardian.co.tt/opinion/tt-divided-by-politics-and-race-6.2.1501787.a83f10527b (Accessed July 18, 2022).

[ref9001] Bahadoorsingh K. (1968). Trinidad electoral politics: the persistence of the race factor [doctoral dissertation]. Indiana University (Accessed January 12, 2022).

[ref3] Banton M. (2012). The color line and the color scale in the 20th century. Ethnic Racial Stud. 35, 1109–1131. doi: 10.1080/01419870.2011.605902

[ref4] Barlow S. A. Fernandez J. R. Sherchan J. S. Monk E. P. Slaughter-Acey J. Sims M. . (2025). Experiences of in-group and out-group skin tone discrimination and their associations with incident cardiovascular disease among African American adults in the Jackson heart study. J. Racial Ethn. Health Disparities, 1–14. doi: 10.1007/s40615-025-02590-8, 40775568 PMC12966262

[ref5] Bernard A. L. (2015). Happiest people alive: an analysis of class and gender in the Trinidad carnival [thesis]. The University of Western Ontario (Canada) electronic thesis and dissertation repository. 3368. Available online at: https://ir.lib.uwo.ca/etd/3368 (Accessed July 1, 2025).

[ref6] Braithwaite L. (1953). Social stratification in Trinidad: a preliminary analysis. Soc. Econ. Stud. 2, 5–175.

[ref7] Branigan A. R. Wildeman C. Freese J. Kiefe C. I. (2017). Complicating colorism: race, skin color, and the likelihood of arrest. Socius Sociol. Res. Dyn. World 3:2378023117725611. doi: 10.1177/2378023117725611

[ref8] Brereton B. (2010). “All ah we is not one:” historical and ethnic narratives in pluralist Trinidad. Glob. South 4, 218–238. doi: 10.2979/globalsouth.4.2.218

[ref10] Caribbean Examinations Council. (2025). CAPE – Caribbean advanced proficiency examination. Available online at: https://www.cxc.org/examinations/cape/ (Accessed March 19, 2025).

[ref11] Cartwright A. (2022). A theory of racialized cultural capital. Sociol. Inq. 92, 317–340. doi: 10.1111/soin.12479

[ref12] Central International Agency. (2025). The world Factbook. Available online at: https://www.cia.gov/the-world-factbook/countries/trinidad-and-tobago/ (Accessed June 20, 2025).

[ref13] Coppin A. Olsen R. N. (1998). Earnings and ethnicity in Trinidad and Tobago. J. Dev. Stud. 34, 116–134. doi: 10.1080/00220389808422524

[ref14] Cordova A. (2008). Methodological note: measuring relative wealth using household asset indicators. AmericasBarom. Insights 6, 1–9.

[ref16] Davenport L. (2020). The fluidity of racial classifications. Annu. Rev. Polit. Sci. 23, 221–240. doi: 10.1146/annurev-polisci-060418-042801

[ref17] Delgado D. J. (2018). My deputies arrest anyone who breaks the law: understanding how color-blind discourse and reasonable suspicion facilitate racist policing. Sociol. Race Ethn. 4, 541–554. doi: 10.1177/2332649218756135

[ref18] Dhillon-Jamerson K. K. (2019). “Marketing marriage and colorism in India” in Race in the marketplace: crossing critical boundaries. eds. Johnson G. D. Thomas K. D. Harrison A. K. Grier S. A. (Cham: Springer International Publishing), 121–136.

[ref19] Dias F. A. (2020). How skin color, class status, and gender intersect in the labor market: evidence from a field experiment. Res. Soc. Stratif. Mobil. 65:100477. doi: 10.1016/j.rssm.2020.100477

[ref20] Dixon A. R. Telles E. E. (2017). Skin color and colorism: global research, concepts, and measurement. Annu. Rev. Sociol. 43, 405–424. doi: 10.1146/annurev-soc-060116-053315

[ref21] Doubeni E. (2017). Understanding colorism through the perceptions and social interactions of African diasporic women [undergraduate honors thesis]. The College of William and Mary. Available online at: https://scholarworks.wm.edu/server/api/core/bitstreams/b01f9e7b-db77-471f-ae5c-85146c6366b6/content (Accessed June 30, 2025).

[ref22] Eberhardt J. L. Davies P. G. Purdie-Vaughns V. J. Johnson S. L. (2006). Looking deathworthy: perceived stereotypicality of black defendants predicts capital-sentencing outcomes. Psychol. Sci. 17, 383–386. doi: 10.1111/j.1467-9280.2006.01716.x, 16683924

[ref23] Ellis N. P. Destine S. (2023). Color capital: examining the racialized nature of beauty via colorism and skin bleaching. Sociol. Compass 17:e13049. doi: 10.1111/soc4.13049

[ref24] Elwin C. (2022). Different forms of media and its influence on ideas of colorism on Afro-Trinidadians [undergraduate thesis]. The University of the West Indies, St. Augustine Campus. Available online at: https://uwispace.sta.uwi.edu/server/api/core/bitstreams/ea3c37f2-fc4e-413f-8bc5-2dc8476d8560/content (Accessed June 30, 2025).

[ref25] Esnard T. (2021). “The education system of Trinidad and Tobago” in The education systems of the Americas: global education systems. eds. Jornitz S. do Parreira M. P. (Cham: Springer), 1–24.

[ref26] Feliciano C. (2016). Shades of race: how phenotype and observer characteristics shape racial classification. Am. Behav. Sci. 60, 390–419. doi: 10.1177/0002764215613401

[ref27] Flores R. Telles E. E. (2012). Social stratification in Mexico: disentangling color, ethnicity, and class. Am. Sociol. Rev. 77, 486–494. doi: 10.1177/0003122412444720 25382861 PMC4222073

[ref28] Gosine M. (1987). “Culture and ethnic participation in a social movement: the case study of the east Indians and the black power movement in Trinidad” in Indians in the Caribbean. ed. Singh B. I. J. (London: Oriental University Press), 217–235.

[ref29] Hall R. E. (2022). Interdisciplinary perspectives on colorism: beyond black and white. New York: Routledge.

[ref30] Hall R. E. Mishra N. (2024). The Routledge international handbook of colorism. New York: Routledge.

[ref31] Hill M. E. (2002). Race of the interviewer and perception of skin color: evidence from the multi-city study of urban inequality. Am. Sociol. Rev. 67, 99–108. doi: 10.1177/000312240206700105

[ref32] Hordge-Freeman E. (2015). The color of love: racial features, stigma, and socialization in black Brazilian families. Austin, TX: University of Texas Press.

[ref33] Hunter M. L. (2002). “If you're light you're alright” light skin color as social capital for women of color. Gend. Soc. 16, 175–193. doi: 10.1177/0891243202016002003, 41467230

[ref34] Hunter M. L. (2007). The persistent problem of colorism: skin tone, status, and inequality. Sociol. Compass 1, 237–254. doi: 10.1111/j.1751-9020.2007.00006.x

[ref35] Hunter M. L. (2011). Buying racial capital: skin bleaching and cosmetic surgery in a globalized world. J. Pan Afr. Stud. 4, 142–165. Available online at: https://www.jpanafrican.org/docs/vol4no4/HUNTER%20Final.pdf

[ref37] Hunter M. L. (2021). “Colorism and the racial politics of beauty” in The Routledge companion to beauty politics. ed. Craig M. L. (London: Routledge), 85–93.

[ref38] Jablonski N. G. (2021). Skin color and race. Am. J. Phys. Anthropol. 175, 437–447. doi: 10.1002/ajpa.24200, 33372701 PMC8247429

[ref39] Keith V. M. Herring C. (1991). Skin tone and stratification in the black community. Am. J. Sociol. 97, 760–778. doi: 10.1086/229819

[ref40] Kelly M. D. A. (2020). Examining race in Jamaica: how racial category and skin color structure social inequality. Race Soc. Probl. 12, 300–312. doi: 10.1007/s12552-020-09287-z

[ref41] Kelly M. D. A. (2022). Racial inequality in the anglophone Caribbean: comparing the cases of Jamaica and Trinidad and Tobago. J. Ethn. Migr. Stud. 49, 125–1153. doi: 10.1080/1369183X.2022.2044767

[ref42] Kelly M. D. A. (2023). The changing terrain of racial inequality in Trinidad and Tobago. Res. Soc. Stratif. Mobil. 86:100826. doi: 10.1016/j.rssm.2023.100826

[ref43] Kelly M. D. A. (2024). Skin color and socioeconomic inequality: the persistence of colorism among Black Jamaicans. Ethnic Racial Stud. 49, –265. doi: 10.1080/01419870.2024.2399717

[ref44] Kelly M. D. A. (2025). Mapping racial fluidity over time in Jamaica. Socius Sociol. Res. Dyn. World 11:23780231251362946. doi: 10.1177/23780231251362946

[ref45] Khadan J. Ruprah I. J. (2024). “Introduction: is Trinidad and Tobago unique?” in Unraveling race, politics, and gender in Trinidad and Tobago’s economic development (Cham: Palgrave Macmillan), 1–34.

[ref46] Khan A. (2009). “Caucasian, coolie, black, or white?” in Shades of difference: why skin color matters. ed. Glenn E. N. (Stanford, CA: Stanford University Press), 95–113.

[ref47] Kim C. J. (1999). The racial triangulation of Asian Americans. Polit. Soc. 27, 105–138. doi: 10.1177/0032329299027001005

[ref48] Kim C. J. (2018). Are Asians the new blacks?: affirmative action, anti-blackness, and the ‘sociometry’ of race. Du Bois Rev. Soc. Sci. Res. Race 15, 217–244. doi: 10.1017/S1742058X18000243

[ref53] Livesay D. (2018). Children of uncertain fortune: mixed-race Jamaicans in Britain and the Atlantic family, 1733–1833. Chapel Hill: University of North Carolina Press.

[ref54] Maddox K. B. (2004). Perspectives on racial phenotypicality bias. Personal. Soc. Psychol. Rev. 8, 383–401. doi: 10.1207/s15327957pspr0804_4, 15582860

[ref55] Maddox K. B. Gray S. A. (2002). Cognitive representations of Black Americans: reexploring the role of skin tone. Personal. Soc. Psychol. Bull. 28, 250–259. doi: 10.1177/0146167202282010

[ref56] Maddox K. B. Perry J. M. Pagan J. (2022). Cues and categories: revisiting paths to racial phenotypicality bias. Soc. Personal. Psychol. Compass 16:e12699. doi: 10.1111/spc3.12699

[ref57] McCree R. (2015). “Race, color and class in Caribbean society” in Routledge international handbook of race, class, and gender. ed. Jackson S. A. (London: Routledge), 233–239.

[ref58] McNamee L. (2020). Colonial legacies and comparative racial identification in the Americas. Am. J. Sociol. 126, 318–353. doi: 10.1086/711063

[ref59] Ministry of the Attorney General and Legal Affairs. (2016). Laws of Trinidad and Tobago. Equal opportunity act. Available online at: https://rgd.legalaffairs.gov.tt/laws2/alphabetical_list/lawspdfs/22.03.pdf (Accessed February 16, 2022).

[ref60] Monk E. P. Jr. (2015). The cost of color: skin color, discrimination, and health among African Americans. Am. J. Sociol. 121, 396–444. doi: 10.1086/682162, 26594713

[ref61] Monk E. P. Jr. (2016). The consequences of “race and color” in Brazil. Soc. Probl. 63, 413–430. doi: 10.1093/socpro/spw014

[ref62] Monk E. P. Jr. (2021a). The unceasing significance of colorism: skin tone stratification in the United States. Daedalus 150, 76–90. doi: 10.1162/daed_a_01847

[ref63] Monk E. P. Jr. (2021b). Colorism and physical health: evidence from a national survey. J. Health Soc. Behav. 62, 37–52. doi: 10.1177/0022146520979645, 33426926

[ref64] Monk E. P. Jr. (2022). Inequality without groups: contemporary theories of categories, intersectional typicality, and the disaggregation of difference. Sociol Theory 40, 3–27. doi: 10.1177/07352751221076863

[ref65] Monk E. P. Jr. Esposito M. H. Lee H. (2021). Beholding inequality: race, gender, and returns to physical attractiveness in the United States. Am. J. Sociol. 127, 194–241.

[ref66] Munasinghe V. P. (2018). Callaloo or tossed salad? East Indians and the cultural politics of identity in Trinidad. Ithaca and London: Cornell University Press.

[ref67] Munasinghe V. P. (2019). “The cunning of multiculturalism: a perspective from the Caribbean” in Multiculturalism in the British commonwealth. eds. Ashcroft R. T. Bevir M. (Berkeley, CA: University of California Press), 212–228.

[ref68] Nettleford R. M. (1998). Mirror mirror: identity, race, and protest in Jamaica. Kingston, Jamaica: Kingston Publishers.

[ref69] Omi M. Winant H. (2018). Racial formations in the United States: from the 1960s to the 1990s. New York: Routledge.

[ref70] Painter M. A. II Holmes M. D. (2023). Persistent skin tone and wealth stratification among new immigrants in the United States. Res. Soc. Stratif. Mobil. 83:100766. doi: 10.1016/j.rssm.2023.100766

[ref71] Painter M. A. II Noy S. Holmes M. D. (2019). Skin tone and asset inequality in Latin America. J. Ethn. Migr. Stud. 46, 3892–3919. doi: 10.1080/1369183X.2019.1592881, 41307611

[ref72] Pedulla D. S. (2018). How race and unemployment shape labor market opportunities: additive, amplified, or muted effects? Soc. Forces 96, 1477–1506. doi: 10.1093/sf/soy002

[ref73] Persaud A. (2024). From sugar to shop: the organic rise of Indian shopkeepers in colonial Trinidad. Bus. Hist. Rev. 98, 921–952. doi: 10.1017/S0007680525000017

[ref74] Premdas R. R. Bangura Y. (2006). “Ethnic conflict, inequality and public sector governance in Trinidad and Tobago” in Ethnic inequalities and public sector governance. ed. Bangura Y. (London: Palgrave MacMillian), 98–119.

[ref9002] Rajkumar F. A. (2006). Ethnicity and economy: the Portuguese, Chinese and Syrian/Lebanese in Trinidad 1945–1981 [doctoral dissertation]. University of the West Indies (Accessed January 02, 2022).

[ref75] Rakhal-Fraser T. (2022a). A dangerous political dead end—what’s ahead? Available online at: https://guardian.co.tt/article/a-dangerous-political-dead-endwhats-ahead-6.2.1505301.236606ac94 (Accessed July 18, 2022).

[ref76] Rakhal-Fraser T. (2022b). Beneath the surface of the corruption blame game. Available online at: https://guardian.co.tt/article/beneath-the-surface-of-the-corruption-blame-game-6.2.1502042.33b0af4bab (Accessed July 18, 2022).

[ref77] Rampersad R. (2012). “Interrogating pigmentocracy: the intersections of race and social class in the primary education of afro-Trinidadian boys” in Intersectionality and race in education. eds. Bhopal K. Preston J. (New York: Routledge), 57–75.

[ref78] Rampersad S. (2022). Kamla’s slave-master comment sparks outrage. Available online at: https://guardian.co.tt/news/kamlas-slavemaster-comment-sparks-outrage-6.2.1501822.74e3d60b9c (Accessed July 18, 2022).

[ref79] Reddy M. (2011). Challenging democracy: ethnicity in postcolonial Fiji and Trinidad. Nationalism Ethnic Polit. 17, 182–202. doi: 10.1080/13537113.2011.575314

[ref81] Roth W. D. Solís P. Sue C. A. (2022). Beyond money whitening: racialized hierarchies and socioeconomic escalators in Mexico. Am. Sociol. Rev. 87, 827–859. doi: 10.1177/00031224221119803

[ref82] Ryabov I. (2016). Educational outcomes of Asian and Hispanic Americans: the significance of skin color. Res. Soc. Stratif. Mobil. 44, 1–9. doi: 10.1016/j.rssm.2015.11.001

[ref9003] Ryan S. D. (1991). Social and occupational stratification in contemporary Trinidad and Tobago. St. Augustine, Trinidad: University of the West Indies, Institute of Social and Economic Research.

[ref83] Ryan S. Greene E. Harwood J. (1979). The confused electorate: a study of political attitudes and opinions in Trinidad and Tobago. St. Augustine, Trinidad: University of the West Indies, Institute of Social and Economic Research.

[ref84] Ryan S. Stewart T. (1995). The black power revolution 1970: a retrospective. St. Augustine, Trinidad: University of the West Indies, Institute of Social and Economic Research.

[ref85] Saperstein A. Penner A. M. (2012). Racial fluidity and inequality in the United States. Am. J. Sociol. 118, 676–727. doi: 10.1086/667722

[ref86] Schimanski C. Chagalj C. Ruprah I. (2018). Race-based educational, occupational and industry segregation and wages gaps in Trinidad and Tobago (Inter-American Development Bank working paper no. 9237). doi: 10.18235/0001380

[ref87] Sio A. A. (1976). Race, color, and miscegenation: the free colored of Jamaica and Barbados. Caribb. Stud. 16, 5–21.

[ref88] Sriskandarajah D. (2005). Development, inequality and ethnic accommodation: clues from Malaysia, Mauritius and Trinidad and Tobago. Oxf. Dev. Stud. 33, 63–79. doi: 10.1080/13600810500099675

[ref89] Stewart N. (2004). Race and color in Trinidad and Tobago (Trinidad & Tobago News Bulletin). Available online at: http://www.trinidadandtobagonews.com/forum/webbbs_config.pl?md=read;id=1467 (Accessed July 6, 2025).

[ref90] Stone C. (1972). Stratification and political change in Trinidad and Jamaica (No. 26). Beverly Hills, CA: SAGE Publications.

[ref91] Strmic-Pawl H. V. Gonlin V. Garner S. (2021). Color in context: three angles on contemporary colorism. Sociol. Race Ethn. 7, 289–303. doi: 10.1177/23326492211012532

[ref92] Tate S. (2007). Black beauty: skin, hair, and anti-racist aesthetics. Ethnic Racial Stud. 30, 300–319. doi: 10.1080/01419870601143992

[ref93] Telles E. (2014). Pigmentocracies: ethnicity, race, and color in Latin America. Chapel Hill: UNC Press Books.

[ref94] Telles E. E. Bailey S. R. Davoudpour S. Freeman N. C. (2025). Racial inequality in Latin America. Oxf. Open Econ. 4, 200–218. doi: 10.1093/ooec/odae022

[ref95] Telles E. E. Flores R. (2013). Not just color: whiteness, nation, and status in Latin America. Hisp. Am. Hist. Rev. 93, 411–449. doi: 10.1215/00182168-2210858

[ref96] Telles E. E. Paschel T. (2014). Who is black, white, or mixed race? How skin color, status, and nation shape racial classification in Latin America. Am. J. Sociol. 120, 864–907. doi: 10.1086/679252, 25848671

[ref97] Vijaya R. M. (2019). “The new economics of colorism in the skin whitening industry: case of India and Nigeria” in Race in the marketplace: crossing critical boundaries. eds. Johnson G. D. Thomas K. D. Harrison A. K. Grier S. A. (Cham: Springer International Publishing), 227–244.

[ref98] Wade P. (2012). Skin color and race as analytic concepts. Ethnic Racial Stud. 35, 1169–1173. doi: 10.1080/01419870.2011.632428

[ref99] Williams E. (1962). History of the people of Trinidad and Tobago. Buffalo, NY: EWORLD Inc.

